# Trends in Precarious Employment in Sweden 1992–2017: A Social Determinant of Health

**DOI:** 10.3390/ijerph191912797

**Published:** 2022-10-06

**Authors:** Theo Bodin, Nuria Matilla-Santander, Jenny Selander, Per Gustavsson, Tomas Hemmingsson, Gun Johansson, Johanna Jonsson, Katarina Kjellberg, Bertina Kreshpaj, Cecilia Orellana, Eskil Wadensjö, Maria Albin

**Affiliations:** 1 Unit of Occupational Medicine, Institute of Environmental Medicine (IMM), Karolinska Institutet, 11365 Stockholm, Sweden; 2 Center for Occupational and Environmental Medicine, Stockholm Region, 11365 Stockholm, Sweden; 3 Department of Public Health Sciences, Stockholm University, 10691 Stockholm, Sweden; 4 Section of Epidemiology, Department of Public Health, University of Copenhagen, 1353 Copenhagen, Denmark; 5 Swedish Institute for Social Research, Stockholm University, 10691 Stockholm, Sweden

**Keywords:** temporary employment, non-standard employment, labor market, employment quality, income, unionization

## Abstract

The aim of this study was to identify trends in precarious employment in the Swedish workforce from 1992 to 2017. This is a repeated cross-sectional study, analyzing the total working population aged 16–75 in Sweden at five-year intervals. We used version 2.0 of the Swedish Register-based Operationalization of Precarious Employment, covering the following dimensions: employment insecurity, income inadequacy, lack of rights and protection. The proportion in precarious employment increased from 9.7 to 12% between 1992 and 2017, a relative increase of 24%. The prevalence was higher among those of lower age, of low education, and immigrants. Differences between sexes converged, and there were slightly more precarious men than women in 2017. The relative increase was most pronounced among men, especially those with low educational attainment and of European origin. The increasing proportion of precarious employees is a clear challenge to the tripartite Nordic model, which requires sufficient trade-union bargaining power.

## 1. Introduction

The ever-changing economic environment in a context of global competition and expansion of financial capitalism has increased the demand from businesses to adopt more non-standard forms of employment and ways of organizing work [1]. Combined with a general weakening of trade unions and changes in labor laws, a variety of non-standard forms of employment have emerged, facilitated by advances in information and communications technology (ICT).

Non-standard forms of employment include temporary and part-time work as well as temporary agency work, but also newer forms of employment, which are very far from the traditional standard of fulltime employment with benefits, such as zero-hour contracts, “gig” work, platform work, multi-party employment, dependent self-employment, or atypical work [2,3,4,5,6].

Data from Europe show that these non-standard forms of employment are increasing. For instance, fewer than six in ten employees have an open-ended contract, and the trend is decreasing in the European Union [2]. In the case of Sweden, atypical employment is defined by the Swedish Labour Policy Council Report as those with any of the following characteristics: not covered by a collective bargaining agreement, having temporary employment, being employed by a temporary staffing agency/self-employed, having one’s own company, holding multiple jobs, or working in the informal sector. This demographic is estimated to be around 35–39% of the Swedish workforce [7]. Globally, and also in the case of Sweden, women, young people, and migrants are especially likely to be in non-standard forms of employment [8].

This gradual transformation of the so-called standard employment relationship into non-standard and atypical employment has bolstered the rise of precarious employment (Bosch, 2004). Further, the rise of non-standard forms of employment may also increase the risk of normalizing precarious employment relationships [9]. 

Precarious employment arrangements can be viewed as a multidimensional construct to capture the complexity of multiple employment arrangement disadvantages, covering employment insecurity (such as being temporarily employed, underemployed, holding multiple jobs), income inadequacy (low income levels or income volatility), and lack of rights and protection (being covered by unions, lack of in-work benefits or social security benefits, and access to or power to exercise workplace rights) as a whole [10]. 

Certain types of non-standard employment are more common in some groups of the population and countries. An in-depth analysis of temporary employment in Sweden shows that within this group of temporary employees, there has been a shift from relatively long fixed-term contracts to more casual forms of employment, such as on-demand employment [11]. In Sweden, a majority of temporary workers tend to remain in this type of employment arrangement or alternate between periods of unemployment, while in other countries, such as Denmark and the Netherlands, holding a temporary job increases the probability of moving into permanent employment [7,12]. In Sweden, only one in ten workers transferred from a temporary to permanent contract within nine months during 2008–2015. For those working as day laborers only, the rate was 1.8% [7]. 

While deregulatory strategies of public authorities are significant drivers of the increase of precarious employment, labor market polarization is still contested [13]. Regarding skills and income, there is an undisputable growth at the top of the labor market, with more people enjoying high salaries in jobs requiring high skills [14]. The changes in the middle and bottom are more debated [15]. In the case of Sweden, although there has been a relative wage polarization, with an increase of jobs in both the highest and lowest income quintiles [16], there has also been a general upgrade across the board with regards to qualifications due to a domestic educational revolution led by women over the last half-century [17]. This is important, as low-skilled workers are particularly affected by cyclical economic downturns and are at higher risk of unemployment and precarious employment conditions [18]. The up-skilling of the labor force that has taken place, especially since the 1990s, has made Sweden more resilient, which was reflected in the very minor impact on the labor market by the great recession of 2008–2010. This is also reflected in the struggles of many OECD countries with jobless growth and stagnating real wages, while Sweden has had one of the highest real-wage growths in the OECD over the last decade [19].

Nonetheless, it is clear that the world of work has undergone profound changes that may have altered the quality of employment relationships in recent decades. Still, the trends have become much more difficult to track due to the imploding response rates to the Labor Force Survey (LFS). Representatives from Statistics Sweden have expressed concerns that labor statistics might have become unreliable [20]. Participation is known to be lower among young people with low levels of education, people with low incomes, and people with foreign backgrounds, as well as among those who have temporary employment or part-time employment or who areself-employed [21]. Thus, our novel approach to operationalize precarious employment arrangements as a multidimensional construct in registers [22] could be used to produce reliable results over time, going beyond the bias of survey data and simplifications of single variables such as income or education.

These changes in the world of work pose considerable challenges for employment quality, occupational safety and health regulations, work environment, and the health and well-being of workers. In our previous studies, we have used our operationalization of precarious employment arrangements to show increased risks for mental [23] and cardiovascular disorders [24], as well as differential health impacts, according to sex and origin [25]. 

Moreover, the COVID-19 pandemic has also revealed that existing public policies to protect workers lag behind the rapid transformations of employment arrangements. For instance, between April and December 2020, only a third of the registered unemployed in Sweden received unemployment benefits [26]. A better monitoring of precarious employment would likely help to prevent gaps in the social protection systems and therefore the health impact of precarious employment.

Available statistics and trends on precarious employment tend to be based on uni-dimensional indicators, such as the proportion of temporary workers. As mentioned before, precarious employment is a very complex phenomenon. Therefore, several indicators of employment quality are required to fully capture the prevalence and the effects of precarious employment and non-standard forms of employment.

In consideration of this background, research into the trends of precarious employment is needed in order to monitor increases in this social determinant of health and determine which groups of the working population are more affected. Therefore, the aim of this study was to identify trends in precarious employment in the Swedish workforce from 1992 to 2017.

## 2. Materials and Methods

### 2.1. Study Design and Population

This is a repeated cross-sectional study, analyzing the total working-age population (16–75 years) in Sweden at five-year intervals, including 1992, 1997, 2002, 2007, 2012, and 2017. To be included in the study, individuals in the working population had to have had an income from work or active business activities of more than 100 SEK (~10 EUR) in the year studied, as well as an employer (this shows that they have been working during the year). We excluded persons receiving disability pension, study grants (received from high school until university), or old-age pension in the year analyzed (to avoid the misclassification of these individuals into precarious employment). Further, we excluded duplicate entries of individuals using the personal ID number.

### 2.2. Data Source

We used the Longitudinal Integration Database for Health Insurance and Labor Market Studies (LISA) maintained by Statistics Sweden. LISA consists of microdata from 1990 and onwards on residents aged 16 or older registered in Sweden on the 31st of December of each year. The database contained all individual variables needed for the study. We also downloaded official seasonally adjusted unemployment rates for the years of interest from the website of Statistics Sweden.

### 2.3. Variables

Precarious Employment

We used a measurement of precariousness based on registry data as previously developed [22]. Briefly, this measurement consists of five items covering the three dimensions of precarious employment as identified previously in a systematic review [10]: a) employment insecurity, b) income inadequacy, and c) lack of rights and protection. This definition summarizes four decades of empirical research and theoretical frameworks defining precarious employment as a multidimensional construct at the level of the employment arrangement [27,28,29,30].

Each individual was assigned a score for each item, resulting in a summative score ranging from −9 to 2 (Table 1). The cutoff for Precarious Employment was <−3 throughout the whole study period. A detailed description and discussion regarding the operationalization can be found in a previous paper [22].

We describe the trends of self-employment with and without employees separately from the precarious employment trends. This is done to avoid a misclassification of the self-employed into precarious employment, as there is limited information available for measuring the quality of self-employment. 

### 2.4. Covariates

In stratified analysis, we used the variables for age (10-year groups), sex (men/women), country of birth (six groups: Sweden and the Nordic Countries, Europe (including the Soviet Union) and Oceania, North America, South America, Asia, and Africa), educational level (primary and lower secondary ~9 years, upper secondary ~3 years, and post-secondary ≥2 years), sector (public/private), employer size (number of registered employees during the year, five groups: 1, 2–9, 10–49, 50–249, ≥250), occupational group (1-digit level of SSYK (the Swedish classification of occupations), i.e., ten groups: Military Personnel; Managers; Professions Requiring In-Depth University Qualifications; Professions Requiring University Qualifications or Equivalent; Administration and Customer Service; Service, Care and Sales Work; Agriculture, Horticulture, Forestry and Fishing; Construction and Manufacturing; Mechanical Manufacturing and Transport and More; Professions Requiring Shorter Education or Introduction).

### 2.5. Statistical Methods

We used basic computations of proportion for each year included in the study, stratified separately by the covariates above. As the data (with very few exceptions) reflect the real N of the total working population, neither samples, statistical testing, nor calculation of confidence intervals were applicable. Regional changes of the proportion of workers in precarious employment were also explored using the county of residence codes. Data management and analyses were performed with Stata version 16 (StataCorp, College Station, TX, USA). Tables and figures were done using Microsoft Excel 365. The syntax is open and free to download and modify via GitHub.

## 3. Results

### 3.1. Participants

After excluding students, the retired, disability pensioners, and duplicates, we obtained the following numbers for analysis at each time-point: N = 3,831,709 in 1992, N = 3,609,559 in 1997, N = 3,741,235 in 2002, N = 3,826,874 in 2007, N = 4,009,764 in 2012, and N = 4,197,900 in 2017.

### 3.2. Descriptive Data 

Sociodemographic characteristics of the study population can be found in Table 2. Some major shifts in the distribution of these characteristics took place during this long period in time. The proportion in the working population with post-secondary education increased by approximately 50% for men while doubling for women between 1992 and 2017. Small (10–49) and medium-sized (50–250) companies increased their share of the workforce, while public sector employment decreased substantially. The latter took place primarily during the crisis in the 1990s. Another important shift is that the proportion of immigrants in the working population increased three to four times and comprised 17% of the total in 2017. The mean age of the working population increased by approximately 1 year for men and 2 years for women. 

### 3.3. Main Results

**Figure 1:**
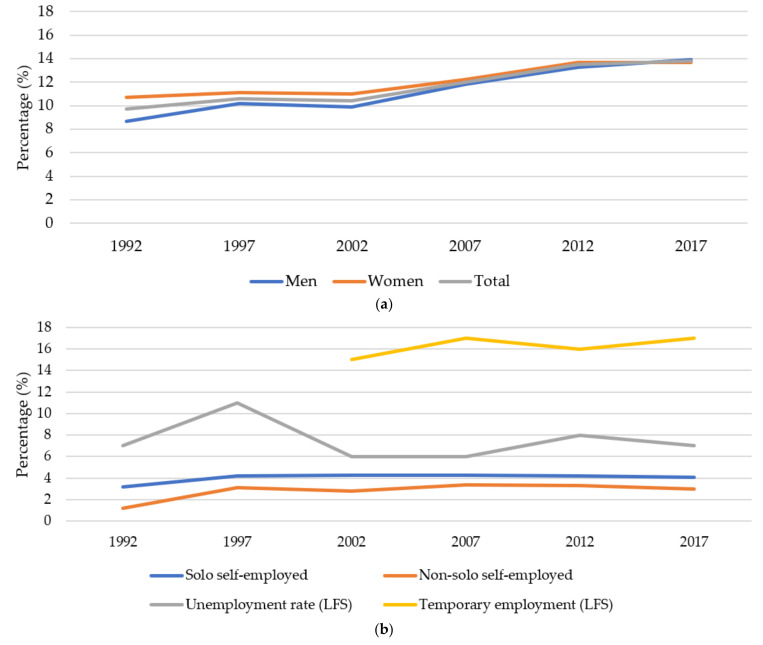
Trends in precarious employment, self-employment, and unemployment in Sweden, 1992–2017. Note: LFS (Labor Force Survey). (**a**) Trends in precarious employment. (**b**) Trends in self-employment, unemployment and temporary employment.

Precarious employment score (PE score <−3) increased from 9.7 to 12% between 1992 and 2017, a relative increase of 23.7% (Figure 1, Table 3). The largest increase took place between 2002 and 2012. LFS data on temporary employment available from 2002 show very little increase overall and none over the last 10 years of the study.

An increased proportion of precariously employed was found across most strata, except for a decline in the public sector, although that curve pointed upwards once again in the last five years of the study.

The stratified analysis gave a more nuanced picture, showing that proportions of men and women in precarious employment were similar in 2017, but had started from a lower level in 1992 for men. There was a sharp increase in precarious employment during the 1990s crisis, 1992–1997, for men, but in the following 20 years, the trend continued—albeit at a slower pace.

In 2017, precarious employment was, with a narrow margin, more common among men than women for the first time. Except for the youngest age group, men had a much worse overall trajectory than women. Interestingly, men from Sweden/other Nordic countries and Europe/Oceania had 29.6%, 38.7% increases in precarious employment, respectively. This was a higher relative increase than for men from other parts of the world during 1992–2017. Also, looking at the 1997–2017 period, Swedish and Nordic men had a higher relative increase than men from other parts of the world. European men were still better-off than non-Europeans in 2017, but the gap had decreased. 

Regarding self-employment, both forms (with and without employees) increased, being higher among men (Tables S1 and S2).

**Figure 2:**
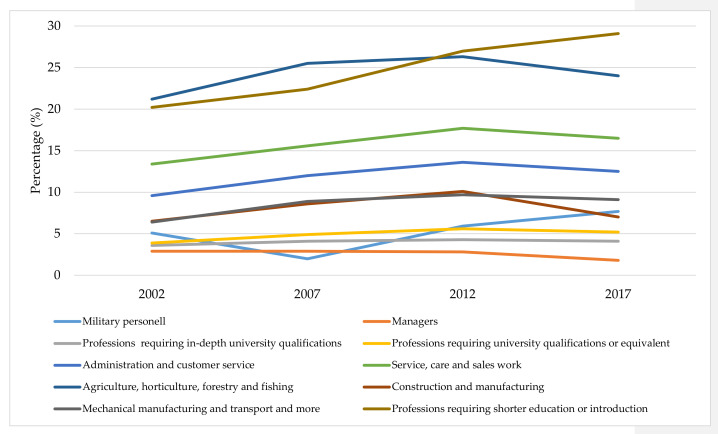
Trends in precarious employment in Sweden by occupational group (1-digit level), 1992–2017.

Analyzing occupational groups, we found that occupations with low requirements on educational attainment were the most affected by an increase in precarious employment but with a stable or decreasing trend between 2012 and 2017. Because of limitations in occupational code data, an analysis could only be done for 2002–2017 (Figure 2).

**Figure 3:**
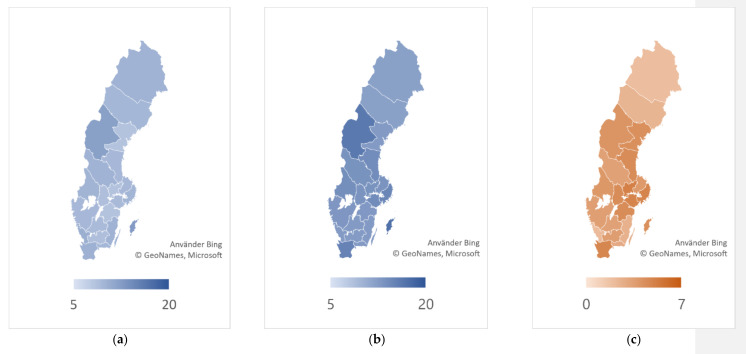
Regional changes in proportion of workers in precarious employment, 2002–2017. (**a**) % of precarious employment 1992; (**b**) % of precarious employment 2017; (**c**) 1992–2017 absolute change of precarious employment.

Precarious employment has increased across all Swedish regions. The large and expansive labor market areas of Stockholm and Skåne had among the highest proportion of precarious workers in 2017 and also saw some of the largest increases in this proportion between 1992 and 2017 (Figure 3). In an analysis of the least-precarious (PE Score > 0), the proportion in most regions was stable or increased after 1997, indicating increased polarization in the labor market (data not shown). 

## 4. Discussion

### 4.1. Key Results

This study found a steady overall increase in precarious employment in Sweden during the years of 1992–2017. Differences across sociodemographic strata show clear patterns according to age, education, and place of birth; the differences between the sexes have converged, and there are now slightly more precarious men than women, both in absolute and relative terms. The trend towards increased precariousness was most pronounced among men, especially those with low educational attainment and of European origin. Further, we found that public-sector workers were less precarious in 2017 than in 1992, although this trend seems to have been broken in the most recent years. Geographical differences could also be identified, indicating an increased polarization in economically expansive regions.

### 4.2. Limitations

Despite the many strengths of this study in covering the whole Swedish population over many years, there are several limitations of the Swedish register data in studying precarious employment. Undeclared work, undocumented immigrants, short-term migrant workers, and posted workers from other EU countries are not included [31]. Statistics Sweden showed in a recent report that 111,000 persons who were not residents in Sweden were paid a salary in 2016, an increase of 131% compared to 1997 [32]. Given that only 31% of these non-resident workers were paid more than 100,000 SEK (~10,000 EUR) and worked short periods, mostly in jobs requiring low qualifications, a vast majority would have been classified as precarious workers were they to be included in the registers, suggesting that this study underestimates the proportion of precarious workers with 2–3 percentage points and underestimates the proportion of precariously employed immigrants. However, one could argue that these individuals are not part of the Swedish population, and when they have lived in Sweden for 3 months to a year, they are likely to be registered. Undeclared workers are of course never detected in the registers, but the informal sector in the Nordic countries is among the lowest in the world at an estimated 3% [7]. In summary, the method applied in this study might not capture 5 percent of the workers, and almost all of these are believed to be precarious. Thus, our study underestimates the increasing trend over the last decades.

Another limitation of the operationalization, as discussed in detail previously, is that it has not been validated empirically yet, primarily because there is no “golden standard” for measuring precarious employment. The sociodemographic characteristics associated with a low precarious score, such as immigrants and those with low education, supports that we are capturing the right population. For the time being, the operationalization must be assessed against the theoretical framework of precarious employment on which it is based. Here, we capture all three dimensions: employment insecurity, income inadequacy, and lack of social and regulatory protection. However, not all aspects of these dimensions are captured. The results from this study fall within the same range as the “atypical employment” measure developed in a report by the labor policy council [7]. The method used in this study shows that a register-based operationalization can be implemented successfully, allowing for more detailed analysis without selection bias. Future etiological studies will determine the usefulness of the operationalization of this social determinant of health. Further, since this study is based in register data, it means that persons who emigrated or died during the year are automatically excluded from the dataset, but appear in the years before, and possibly after in case of re-immigration. Thus, selection bias of the study population can be ruled out.

### 4.3. Interpretation

The structural changes in the labor market during the 25 years covered in this study have had contradictory effects on the proportion of the precariously employed overall. The increase in older workers and the increased educational attainment, especially for women, have probably kept the precarious employment levels lower than if those variables would have been held constant, confirming a general improvement in work–life balance and employment arrangements due to gendered up-skilling, as put forward by others [17,33]. The decrease of employees in the public sector, with simultaneous increase in small- and medium-sized companies, as well as increase in the immigrant working population, are associated with the increase in precarious employment. Although the main drivers for the increase in precarious employment could not be determined by this study, and while there are many political and sociological changes in society that cannot be measured directly, some of these associations point at societal challenges and their relative importance over time. A larger immigrant population increases the proportion of workers in precarious employment as this demographic has difficulties in gaining a strong footing in the labor market [34,35,36]. However, this study shows that in relative terms, most immigrant groups had a slower increase in their proportion of precarious employees than Swedes and Europeans while starting off at a more disadvantaged position. This somewhat contradicts the general notion of an increased racification of the precarious workforce: rather, it has been this way for many decades, but the population has increased in overall size. Still, almost half of the working population born in Africa or Asia were precarious in 2017. 

Further, this study does not consider long-term unemployment and informal work among immigrants. The results in this study also contradict international findings and gendered discourse of precarious employment disadvantaging women [37,38]. Instead, we found that men overall have slowly become as precarious as women, a shift likely driven by the very significant differences in trends regarding educational attainment. Although the self-employed in Sweden consist of both high and low earners and consist of both self-employed and well-off business owners, they lack the same income security and access to social security as permanent employees, and as a growing group, they are important to include. Once again, a multidimensional perspective is needed, as the single dimension of self-employed leads to misclassification.

Further, if the increasing trend in precarious employment continues, which most forecasts indicate, a new approach to organizing precarious workers will be required to maintain this balance of power [39]. 

### 4.4. Health Implications of the Results 

Our results show a steady increase of precarious employment over the years. Precarious employment has been associated with an increased risk of several health outcomes, including mental disorders [40,41,42,43], cardiovascular disorders [24], increased prevalence of musculoskeletal pain in Sweden [44] and in the European Union (including backache and upper and lower muscular pain) [45], and increased prevalence of infectious and respiratory problems [46]. Therefore, the increase in precarious employment observed in our study will implicate a higher burden of disease associated with precarious employment. 

Furthermore, our study also shows differences of precarious employment across sociodemographic strata, especially relevant according to age, education, and place of birth. Since precarious employment has been reported to increase the risk of several health outcomes, and we show that precarious employment is higher among certain sociodemographic groups, this would also have implications for the creation of health inequalities among the Swedish working population. 

### 4.5. Generalizability

The study of precarious employment in public health is intertwined with difficult questions on causality and exposure time. Such questions typically require many years of longitudinal follow-up data, including detailed information on the employment trajectories of often hard-to-reach categories of workers. To date—even on a global level—no single-panel survey is able to provide this kind of data. Although self-reports derived from good-quality survey panels might still provide superior information, contemporary register data have the potential to provide new opportunities. Register data have a number of advantages: (1) they describe entire populations, making it easier to select hard-to-reach groups of workers; (2) they include high-quality objective data; (3) they enable the composition of large time-series at the level of individual workers; (4) they might include links between the employment situation and individual data of high relevance for public health (e.g., registered mortality, disability, medical consumption). Preliminary results from upcoming studies within our research group show very promising results, especially in creating large longitudinal trajectory-based studies and efforts to create similar operationalizations in other countries. Our group welcomes everyone who is interested in developing this methodology, and we are happy to collaborate across borders.

## 5. Conclusions

The increasing proportion of precarious employees in the Swedish labor market is a societal challenge that needs to be addressed forcefully and with a sense of urgency. Lying behind these trends is a complex system of global trends, policy, and demographic changes. It is a clear challenge to the tripartite Nordic model, which requires trade unions to have sufficient bargaining power throughout the labor market to be functional. If the trend continues, which most forecasts indicate, a new approach to organizing precarious workers will be required to maintain this balance of power. To understand this development in a complex sociopolitical and economic environment, a multidimensional construct approach provides a more nuanced picture and insights into recent developments, which opens the route for labor-market and public-health studies of high quality.

## Figures and Tables

**Table 1:** Measurement of precarious employment in the Swedish register data.

Theme	Categories	Score
Contractual relationship insecurity	Directly employed by the employer	0
	Employed by a temp agency °	−1
Contractual Temporariness	Having the same employer for 3 years	0
	Having the same employer for <3 years	−2
Number of employers during the year	1–2 employers	0
	≥3 employers	−1
	≥3 employers in ≥3 sectors	−2
Income in relation to the median salaried income in total working population	>200%	2
	120–200%	1
	80–120%	0
	60–80%	−1
	<60%	−2
Probability of unionization coverage *	>90%	0
	70–90%	−1
	<70%	−2

* Only available for 2002–2014. The values for 1992, 1997, and 2017 were assigned the values for closest year with data available. ° Only available for 2002–2017 (temporary agency employment was regulated for the first time in Sweden in 1993 and first started to be registered with the Swedish Standard Industrial Classification of 2002).

**Table 2:** Sociodemographic characteristics of the Swedish working population over time, every 5 years.

N	1992		1997		2002		2007		2012		2017		1992–2017 Relative Change	
	Men	Women	Men	Women	Men	Women	Men	Women	Men	Women	Men	Women	Men	Women
	2,021,427	1,816,626	1,928,138	1,681,421	2,006,694	1,734,541	2,055,351	1,771,523	2,126,215	1,883,549	2,232,546	1,965,354	12%	8%
**Mean age (sd)**	33.6 (15.2)	33.5 (15.2)	34.9 (14.3)	35.5 (13.9)	35.9 (14.4)	36.4 (14.1)	35.8 (14.8)	36.3 (14.4)	35.5 (15.3)	36.2 (14.8)	35.5 (15.1)	36.3 (14.7)		
**Region of birth**														
Africa	0.4	0.2	0.5	0.3	0.8	0.6	1	0.7	1.4	1.1	2.3	1.8	475.0	800.0
Asia	1.5	1.1	1.9	1.5	3	2.7	4.1	3.7	5.7	5.2	8.1	7.3	440.0	563.6
Europe and Oceania	2.9	2.7	3.2	2.9	3.9	3.9	4.6	4.6	5.5	5.5	6.7	6.7	131.0	148.1
North America	0.2	0.2	0.2	0.2	0.3	0.3	0.3	0.3	0.4	0.4	0.5	0.4	150.0	100.0
South America	0.5	0.5	0.5	0.5	0.7	0.7	0.8	0.8	0.9	0.9	0.9	1	80.0	100.0
Sweden and Nordics	94.5	95.3	93.7	94.6	91.4	92	89.1	89.9	86.1	86.9	81.4	82.8	−13.9	−13.1
**Employers size**														
1	46.6	65.8	50.6	66.5	47.5	62.5	47.2	56.7	50.4	59.3	48.4	56.7	3.9	−13.8
2–9	18.6	31	25.1	36.1	23.6	34.3	26.3	33.8	26.5	34.8	25.9	33.2	39.2	7.1
10–49	8.2	13.9	11.4	17.3	10.8	16	8.9	15.4	9.2	15.8	9.7	14.6	18.3	5.0
50–249	4.6	9.3	6.3	11.6	6.6	11.5	5.5	9.9	5.5	10.1	6.9	11	50.0	18.3
250–	5.9	7.4	5.7	6.5	5.8	6.9	4.3	5.2	5	5.9	6.7	6.7	13.6	−9.5
Sector														
Private	79.2	51.1	75	43.8	77.8	47	79.8	48.4	80.7	51.3	80.2	51.1	1.3	0.0
Public	20.8	48.9	25	56.2	22.2	53	20.2	51.6	19.3	48.7	19.8	48.9	−4.8	0.0
**Educational level**														
Primary	28	22.5	23.4	17.9	19.1	13.6	15.8	10.2	13.2	8.2	11.8	7.2	−57.9	−68.0
Secondary	49.1	51.4	50.9	51.2	52.5	51.1	52.7	48.9	52.7	46.5	51.9	43.2	5.7	−16.0
Post-secondary	22.9	26.1	25.7	30.9	28.5	35.3	31.5	40.9	34	45.4	36.4	49.6	59.0	90.0

**Table 3:** Proportion of workers in precarious employment (PE score <−3) in Sweden, 1992–2017.

	1992		1997		2002		2007		2012		2017		1992–2017 Relative Change		1997–2017 Relative Change	
	Men	Women	Men	Women	Men	Women	Men	Women	Men	Women	Men	Women	Men	Women	Men	Women
**Total**	8.7	10.7	10.2	11.1	9.9	11	11.8	12.2	13.3	13.7	13.9	13.7	59.8	28.0	36.3	23.4
**Age**																
≤24	28.6	28.6	39.2	43.2	38.3	45.7	41.7	51.9	44.6	54.9	44.5	51.3	55.6	79.4	13.5	18.8
25–34	9.2	12.3	11.2	14.3	11.4	14.1	15.4	16.8	17.1	18.4	18	18.9	95.7	53.7	60.7	32.2
35–44	5.9	8.2	7.4	8.6	7	8.6	7.8	8.6	8.4	9.3	10	10	69.5	22.0	35.1	16.3
45–54	4.3	5.8	5.2	5.1	5.4	5.2	6.3	5.6	6.8	6.8	7.1	6.9	65.1	19.0	36.5	35.3
≥55	4	6.3	4	4.6	4.6	4.7	4.9	4.3	5.6	4.9	6.3	5.3	57.5	−15.9	57.5	15.2
**Region of birth**																
Africa	28.6	28.5	31.5	34.1	27.8	28.8	33.4	26.4	34.5	30.1	35.6	32.6	24.5	14.4	13.0	−4.4
Asia	30.2	28.9	36.2	33	32.7	29.8	37.3	28.9	35.4	29.3	35.6	27.8	17.9	−3.8	−1.7	−15.8
Europe and Oceania	14.2	15.7	21.1	22	17.1	19.6	20.9	20.2	20.9	21.2	19.7	20.7	38.7	31.8	−6.6	−5.9
North America	22.8	26.3	26.2	27	22.9	25	22.9	22.8	23.1	24.5	22.4	22.9	−1.8	−12.9	−14.5	−15.2
South America	21.2	22.3	25.8	26	23.6	23.4	24.9	23	23.9	21.9	20.9	19.9	−1.4	−10.8	−19.0	−23.5
Sweden and Nordics	8.1	10.2	9.1	10.2	8.6	9.8	9.8	10.9	10.8	11.9	10.5	11.2	29.6	9.8	15.4	9.8
**Employer Size**																
1	46.6	65.8	50.6	66.5	47.5	62.5	47.2	56.7	50.4	59.3	48.4	56.7	3.9	−13.8	−4.3	−14.7
2–9	18.6	31	25.1	36.1	23.6	34.3	26.3	33.8	26.5	34.8	25.9	33.2	39.2	7.1	3.2	−8.0
10–49	8.2	13.9	11.4	17.3	10.8	16	8.9	15.4	9.2	15.8	9.7	14.6	18.3	5.0	−14.9	−15.6
50–249	4.6	9.3	6.3	11.6	6.6	11.5	5.5	9.9	5.5	10.1	6.9	11	50.0	18.3	9.5	−5.2
≥250	5.9	7.4	5.7	6.5	5.8	6.9	4.3	5.2	5	5.9	6.7	6.7	13.6	−9.5	17.5	3.1
Sector																
Private	8.7	15.4	11.4	18.3	10.8	16.9	9.9	15.6	10.3	15.9	11.2	15.8	28.7	2.6	−1.8	−13.7
Public	9.3	8.2	7	6.1	7	6.2	4.1	4.1	5.2	4.7	6.8	5.3	−26.9	−35.4	−2.9	−13.1
**Educational level**																
Primary	9.2	14.1	10.9	14.4	13.1	17.2	17.2	20.7	20.2	25	25	29.9	171.7	112.1	129.4	107.6
Secondary	9.5	11.5	11.7	12.5	10.6	12.2	12.2	14	13.6	16.1	13.5	15.9	42.1	38.3	15.4	27.2
Post-secondary	5	5.3	5.9	6.5	5.9	6.5	7.5	7.6	8.3	8.2	8.9	8.1	78.0	52.8	50.8	24.6

## Data Availability

Not applicable.

## Supplementary Materials

The following supporting information can be downloaded at https://www.mdpi.com/article/10.3390/ijerph191912797/s1: Table S1: Proportion of solo self-employed individuals in Sweden, 1992–2017; Table S2: Proportion of self-employed with employees in Sweden, 1992–2017.
