# Supplementary material for: Trends in Precarious Employment in Sweden 1992–2017: A Social Determinant of Health
Source: Int J Environ Res Public Health. 2022 Oct 6;19(19):12797. doi: 10.3390/ijerph191912797 (PMC9565988; doi:10.3390/ijerph191912797)
Supplement: Supplementary file 1 [file ijerph-19-12797-s001.zip › ijerph-1920072-supplementary.pdf]

Supplementary Material

Table S1. Proportion of solo self-employed individuals in Sweden 1992–2017.

	1992		1997		2002		2007		2012		2017		Relative Change 1992–2017		Relative Change 1997–2017	
	Men	Women	Men	Women	Men	Women	Men	Women	Men	Women	Men	Women	Men	Women	Men	Women
**Total**	4.5	1.8	5.7	2.5	5.8	2.6	5.5	2.8	5.3	2.9	5.1	3	13.3	66.7	−10.5	20.0
**Age groups**																
≤24	1.1	0.6	1.5	1	1.2	0.8	1	0.9	1.1	1	0.9	0.8	−18.2	33.3	−40.0	−20.0
25–34	3.4	1.4	3.7	1.9	3.5	1.8	3.3	1.9	3.2	2.1	2.8	2	−17.6	42.9	−24.3	5.3
35–44	5.1	1.9	6.3	2.6	6	2.9	5.4	3	5.3	3	5.2	3	2.0	57.9	−17.5	15.4
45–54	5.4	2.1	7.2	2.8	7.4	3	7.1	3.4	6.9	3.7	6.7	3.8	24.1	81.0	−6.9	35.7
≥55	6.7	2.8	7.7	3.3	8.2	3.6	8.2	3.7	7.9	3.6	8	4	19.4	42.9	3.9	21.2
**Country of birth**																
Africa	3.4	1.4	5	1.7	4.5	1.3	3.1	1.2	2.5	1.2	2	1	−41.2	−28.6	−60.0	−41.2
Asia	10.5	2.9	14.8	4.8	12.1	4.3	6.7	3.8	5.1	3.3	4.3	3.1	−59.0	6.9	−70.9	−35.4
Europe and Oceania	6.1	2.7	6.7	3.8	5.9	3.3	5.1	3.1	5.6	3.8	5.1	3.7	−16.4	37.0	−23.9	−2.6
North America	3.8	2.5	5.4	4.3	5.2	4.5	5.4	5	5.8	4.6	5.2	4.2	36.8	68.0	−3.7	−2.3
South America	2	0.7	2.9	1.4	2.9	1.6	2.6	1.8	2.7	2	3.1	2.3	55.0	228.6	6.9	64.3
Sweden and Nordics	4.4	1.8	5.5	2.4	5.6	2.6	5.5	2.8	5.4	2.9	5.3	3	20.5	66.7	−3.6	25
**Educational level**																
Primary	7.2	2.6	8.5	3.4	8.5	3.4	7.7	3.6	6.9	3.6	6	3.5	−16.7	34.6	−29.4	2.9
Secondary	4.1	1.7	5.4	2.5	5.7	2.7	5.4	3	5.3	3.1	5.1	3.1	24.4	82.4	−5.6	24.0
Post-secondary	2	1.1	3.6	1.9	4.2	2.2	4.5	2.5	4.8	2.7	4.9	2.8	145.0	154.5	36.1	47.4

Table S2. Proportion of self-employed with employees in Sweden 1992–2017.

	1992		1997		2002		2007		2012		2017		Relative Change 1992–2017		Relative Change 1997–2017	
	**Men**	**Women**	**Men**	**Women**	**Men**	**Women**	**Men**	**Women**	**Men**	**Women**	**Men**	**Women**	**Men**	**Women**	**Men**	**Women**
**Total**	1.4	1.1	4	2.1	3.7	1.8	5	1.5	4.8	1.6	4.4	1.5	214.3	36.4	10.0	−28.6
**Age groups**																
≤24	0.1	0.1	0.6	0.3	0.4	0.2	0.7	0.3	0.6	0.2	0.5	0.2	400.0	100.0	−16.7	−33.3
25–34	0.7	0.5	2.1	1	2	0.9	3.1	0.9	2.9	0.9	2.3	0.8	228.6	60.0	9.5	−20.0
35–44	1.5	1.1	4.4	2.2	4	1.9	5.8	1.7	5.7	1.8	5.1	1.6	240.0	45.5	15.9	−27.3
45–54	1.9	1.5	5.7	2.8	5.1	2.4	6.7	2	6.7	2.1	6.2	2.1	226.3	40.0	8.8	−25.0
≥55	2.8	1.8	5.7	2.8	5.2	2.5	6	1.9	5.7	1.8	5.5	1.7	96.4	−5.6	−3.5	−39.3
**Country of birth**																
Africa	0.9	0.6	1.8	1	1.9	0.9	2.3	0.6	1.9	0.7	1.3	0.5	44.4	−16.7	−27.8	−50.0
Asia	2.8	1.5	5.4	2.7	5.7	2.6	8.3	2.4	7.5	2.5	5.7	2.1	103.6	40.0	5.6	−22.2
Europe and Oceania	1.7	1.3	3.7	2.4	3	1.7	3.8	1.6	3.8	1.9	3.6	1.9	111.8	46.2	−2.7	−20.8
North America	0.6	0.7	1.7	1.7	1.9	2.3	2.5	1.7	2.2	1.5	2	1.3	233.3	85.7	17.6	−23.5
South America	0.2	0.1	0.9	0.7	1.2	0.8	1.7	0.9	2.2	1.1	2.3	1.1	1050.0	1000.0	155.6	57.1
Sweden and Nordics	1.4	1	4.1	2	3.8	1.8	5	1.5	4.8	1.5	4.4	1.4	214.3	40.0	7.3	−30.0
**Educational level**																
Primary	2.4	2.1	6	3.7	5.6	3.1	6.8	2.2	6.5	2.2	5.7	1.9	137.5	−9.5	−5.0	−48.6
Secondary	1.3	0.9	4	2.1	3.7	1.9	5.1	1.7	5.1	1.7	4.6	1.6	253.8	77.8	15.0	−23.8
Post-secondary	0.5	0.4	2.4	1.1	2.6	1.1	4	1.2	4	1.3	3.6	1.3	620.0	225.0	50.0	18.2
